# Chest pain while gardening: a Stanford type A dissection involving the aortic root extending into the iliac arteries—an uncommon and potentially catastrophic disease process

**DOI:** 10.1186/s12245-019-0237-8

**Published:** 2019-08-30

**Authors:** Gregory M. Taylor, Michael W. Barney, Eric L. McDowell

**Affiliations:** 1 D.O. Assistant Professor of Clinical Emergency Medicine, Indiana University School of Medicine, Ball Memorial Hospital, Department of Emergency Medicine, 2401 W. University Ave, Muncie, IN 47303 USA; 2 D.O. Assistant Clinical Professor, Beaumont Hospital, Botsford Campus, Teaching hospital of Michigan State University, Department of Emergency Medicine, Farmington Hills, MI USA

**Keywords:** Shock, Hypertension, Aortic dissection, Chest pain, Weakness

## Abstract

**Background:**

An aortic dissection is an uncommon and potentially catastrophic disease process that carries with it a high morbidity and mortality. The inciting event is a tear in the intimal lining of the aorta. This allows passage of blood through the tear and into the aortic media, resulting in the creation of a false lumen.

**Case presentation:**

We describe the case of a 71-year-old male with a history of hypertension that suffered a Stanford type A dissection with an intimal flap beginning at the level of the aortic root and extending into the bilateral iliac arteries. His clinical presentation was further complicated by shock, cardiac tamponade, severe coagulopathy, an ischemic right lower extremity, infarction of his thoracic spinal cord, and subacute infarcts secondary to malperfusion and embolic disease. Despite maximal intervention, the patient continued to clinically decline and ultimately died on day 5.

**Conclusion:**

The clinical presentation of an acute aortic dissection is often atypical and mimics other common disease processes. The signs and symptoms largely depend on the extent of the aortic dissection and the presence or absence of malperfusion. With a mortality increasing by 1–2% for every hour until definitive treatment, early recognition and prompt operative intervention are crucial for patient survival.

## Introduction

The incidence of an aortic dissection ranges from 2.6 to 3.5 per every 100,000 patients that survive to reach the hospital [1]. This number is likely underestimated as it does not take into consideration the many patients that die before reaching the hospital.

## Case presentation

A 71-year-old male with a past medical history of hypertension presented to our community emergency department (ED) transported by emergency medical services (EMS) with the chief complaints of chest pain, difficulty breathing, back pain, and weakness of both lower extremities. He had an abrupt onset of chest pain, described as a sharp and stabbing pain, while using his rototiller in the garden, 6 h prior to arrival. His symptoms continued to progress to the point that he was having difficulty breathing and starting to have trouble walking for which EMS was called. Per EMS, they noted rapidly progressing symptoms while en route to the ED. Vitals on arrival to the ED are as follows: 94.5 °F, blood pressure of 91/64 mmHg, heart rate of 109 beats/min, respiratory rate of 22 breaths/min, and 100% pulse oximetry on room air. On physical exam, he was toxic appearing, in acute distress, and diaphoretic. Jugular venous distention was present. Cardiopulmonary exam was notable for tachycardia, distant heart sounds, and the presence of bibasilar rales. His skin/lower extremities were pale, mottled, and he was unable to move them. Carotid pulses were 2+ bilaterally, radial pules 1+ bilaterally, femoral pulses 1+ bilaterally, with absent popliteal pulses. On neurological exam, he had dysarthric speech, left-sided facial droop, and loss of strength and sensation in his lower extremities.

**Fig. 1:**
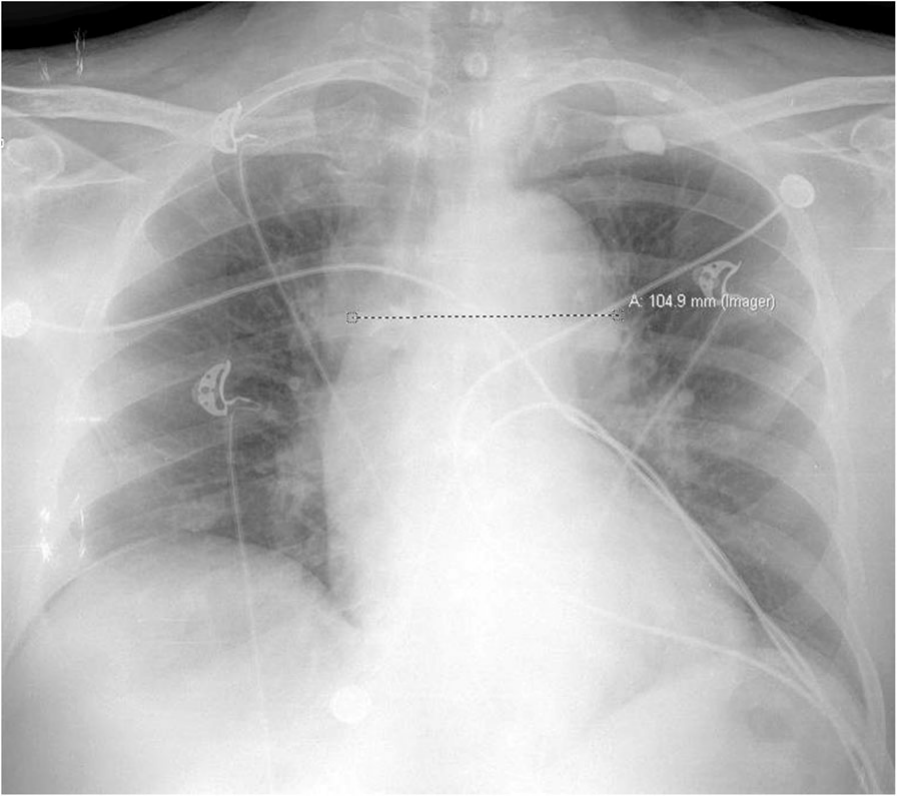
Chest radiography, upright, demonstrating a widened mediastinum at 10.5 cm

The patient was placed on non-invasive positive pressure. Electrocardiography revealed sinus tachycardia with premature atrial complexes, a right bundle branch block, with non-specific ST/T wave changes in the anterior leads. Portable chest radiography taken in an upright position (Fig. 1) demonstrated a widened mediastinum at about 10.5 cm. A bedside point of care cardiac ultrasound revealed a significant pericardial effusion. Computed tomography (CT) of the head without contrast was unremarkable for any acute process. Pertinent laboratory evaluation:

- Hemoglobin of 9.2 (13.5–17 g/dL)
- Arterial blood gas: pH 7.01, pCO2 45, p02 116, HCO3 12
- Initial lactic acid of 8.3 (0.5–2.2 mmol/L) that increased to 14.8
- Blood/urea/nitrogen of 42 (8–22 mg/dL)
- Creatinine of 3.06 (0.6–1.4 mg/dL).

**Fig. 2:**
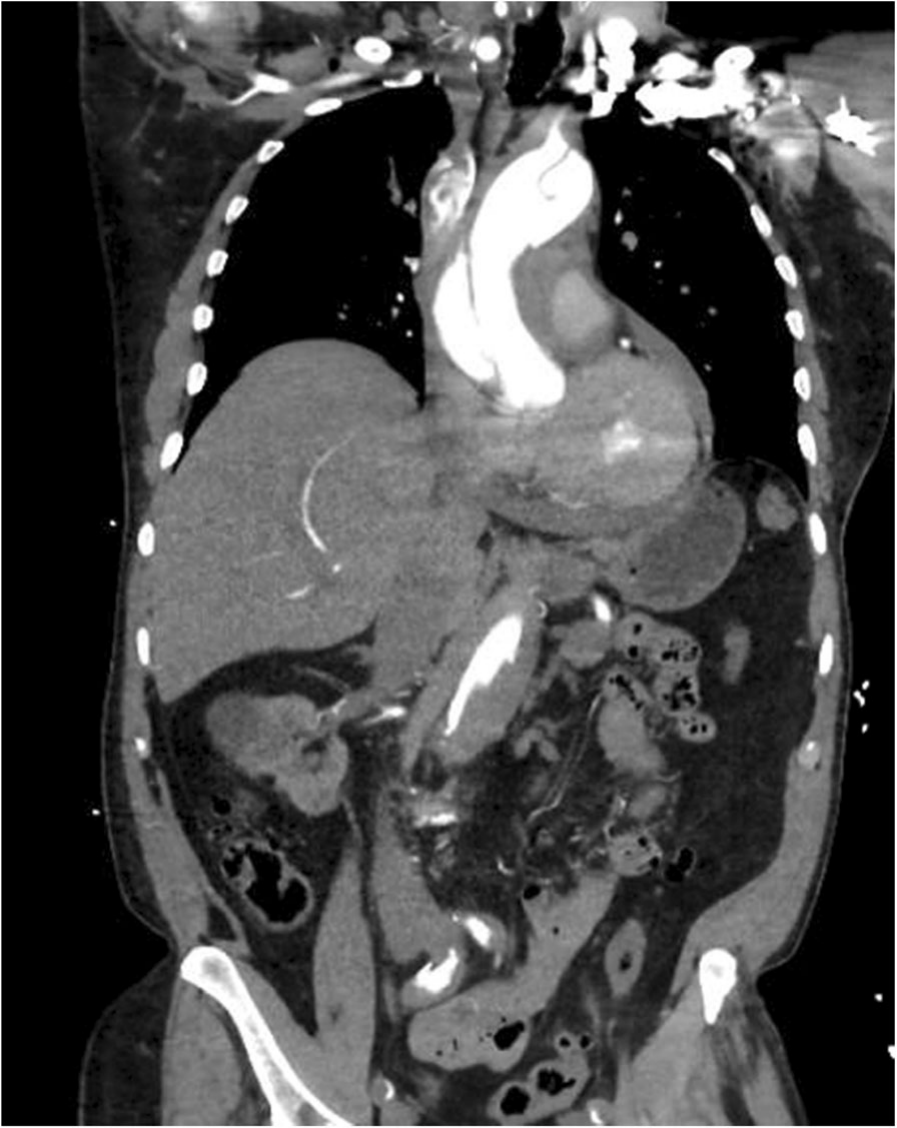
CT angiography of the chest, abdomen, and pelvis with and without intravenous contrast revealing a Stanford type A dissection with an intimal flap seen beginning at the level of the aortic root and extending throughout the course of the thoracic aorta and extending into the abdominal aorta

**Fig. 3:**
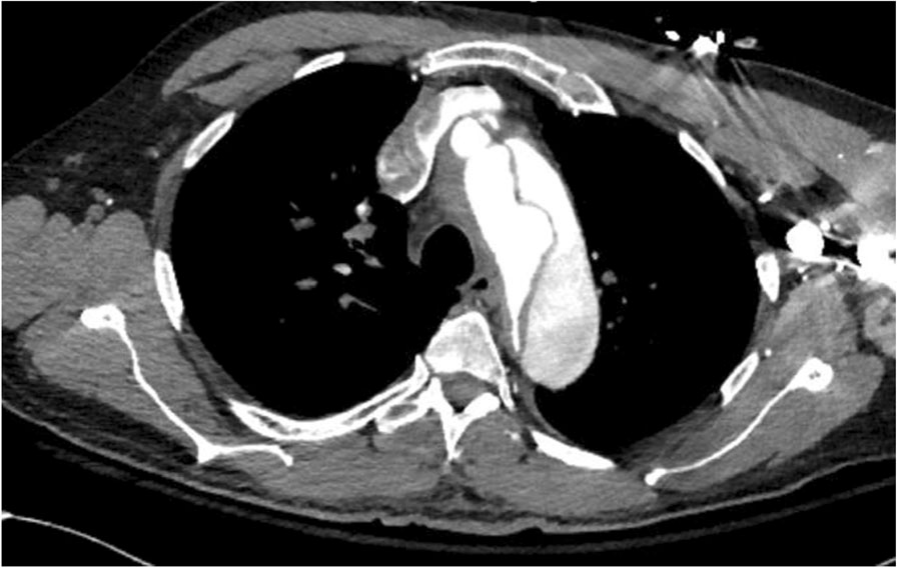
CT angiography of the chest, abdomen, and pelvis with and without intravenous contrast revealing a Stanford type A dissection with an intimal flap seen beginning at the level of the aortic root and extending throughout the course of the thoracic aorta and extending into the abdominal aorta

**Fig. 4:**
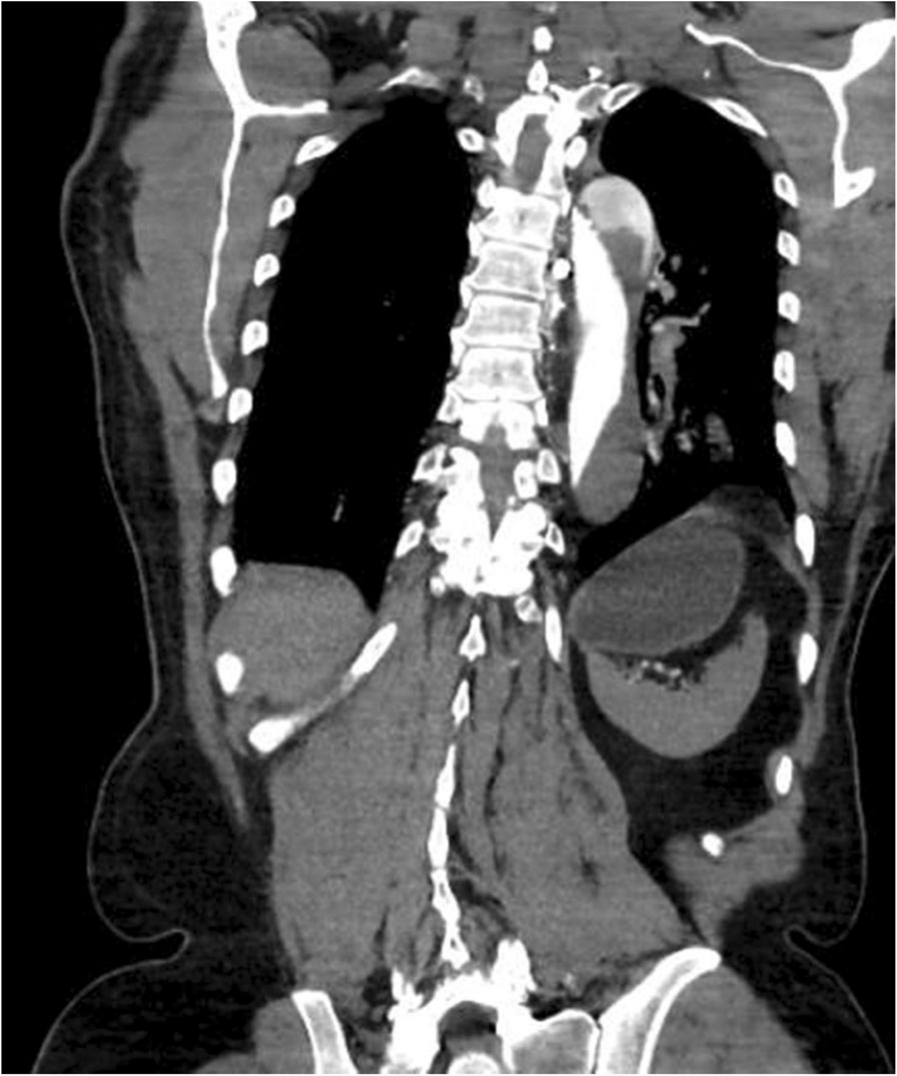
CT angiography of the chest, abdomen, and pelvis with and without intravenous contrast revealing a Stanford type A dissection with an intimal flap seen beginning at the level of the aortic root and extending throughout the course of the thoracic aorta and extending into the abdominal aorta

**Fig. 5:**
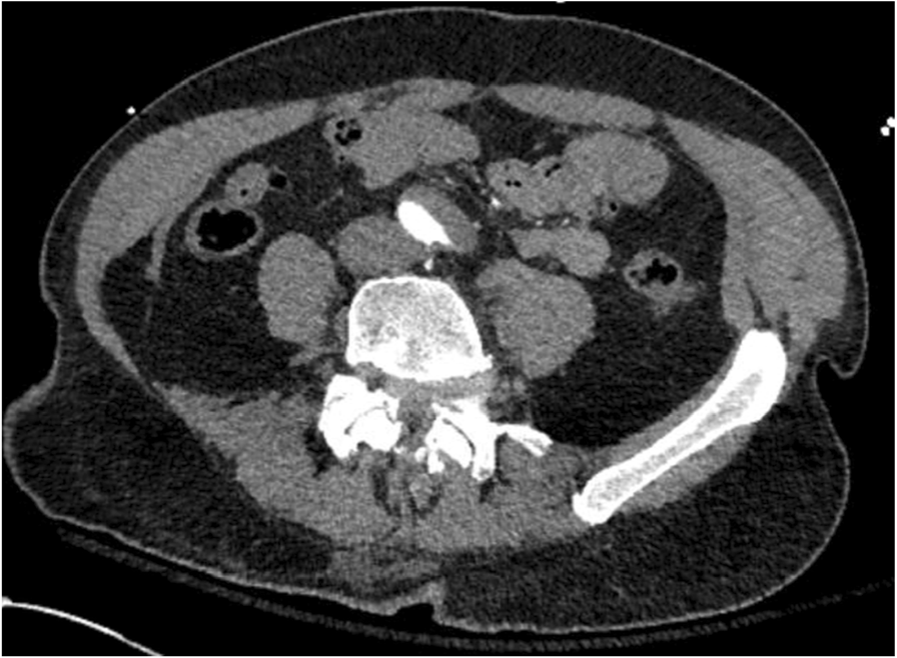
CT angiography demonstrating continued dissection throughout the abdominal aorta to involve the left common iliac artery and bilateral external iliac arteries

**Fig. 6:**
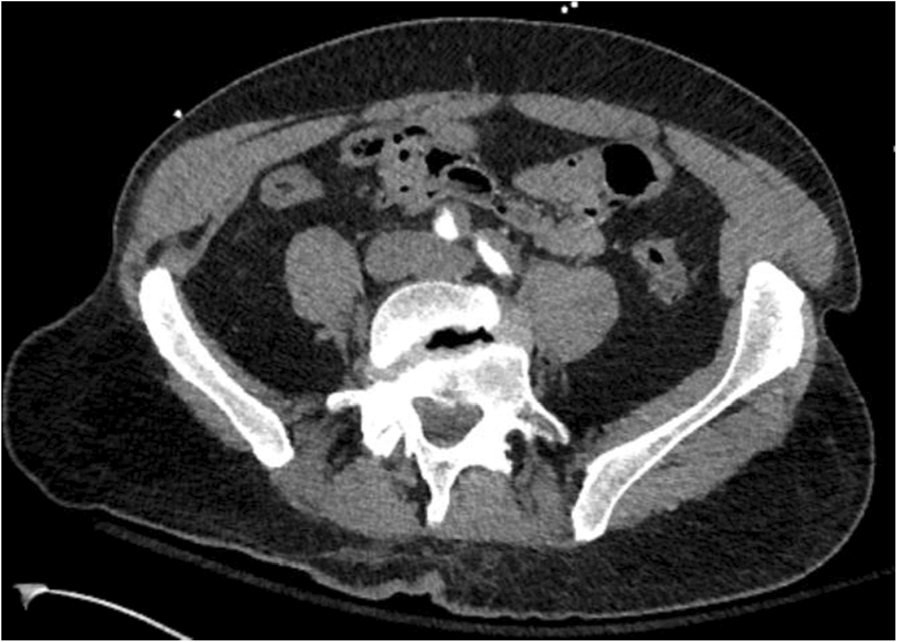
CT angiography demonstrating continued dissection throughout the abdominal aorta to involve the left common iliac artery and bilateral external iliac arteries

CT angiography of the chest, abdomen, and pelvis with and without intravenous contrast revealed a Stanford type A dissection with an intimal tear beginning at the level of the aortic root and extending throughout the course of the thoracic aorta and into the abdominal aorta (Figs. 2, 3, and 4). Additional findings on CT included involvement of the origins of the great vessels (brachiocephalic trunk, left common carotid artery, left subclavian artery). Hemopericardium is visualized with high suspicion for pericardial tamponade. There is dilation of the ascending thoracic aorta measuring up to 5.2 cm. The dissection continues distally (Figs. 5 and 6) to involve the left common iliac artery and bilateral external iliac arteries.

He was transferred emergently to a tertiary center and was taken immediately to the operating room for definitive repair. The patient underwent sternotomy, repair of Stanford type A dissection with right groin cannulation, and repair of the right femoral artery and vein. He arrived in the surgical intensive care unit in guarded condition, his sternotomy was left open secondary to severe coagulopathy intraoperatively, and he was on an epinephrine, norepinephrine, and vasopressin infusion. By day 2, continuous renal replacement therapy (CCRT) was initiated for acute tubular necrosis. Vascular surgery was consulted to evaluate a pulseless right lower extremity status post right femoral artery cannulation for cardiopulmonary bypass. Given the patient had an open sternotomy with five chest tubes with significant output requiring transfusion of multiple blood products throughout the night, vascular surgery could not perform a femoral-femoral bypass or axillofemoral bypass without heparinization.

By day 3, he underwent mediastinal irrigation and sternal closure with cardiothoracic surgery. He was then taken for a right common femoral endarterectomy, iliofemoral and lower extremity embolectomy, and right lower extremity four-compartment fasciotomy secondary to acute ischemia of the right lower extremity. In addition, neurosurgery evaluated the patient because he was not spontaneously moving his extremities after surgery and there was concern that the dissection had caused an infarction of his thoracic spinal cord, resulting in paraplegia. By day 4, he remained stuporous off sedation and on maximum pressor support (norepinephrine infusion at 30 mcg/min, epinephrine infusion at 10 mcg/min, and a vasopressin infusion at 0.04 units/min). He continued to have upper extremity non-epileptic myoclonic jerks secondary to continued renal failure. CT of the head revealed multiple 1 to 2 cm foci consistent with likely subacute infarcts related to embolic disease. By day 5, a family discussion was had regarding a poor neurological prognosis for independent neurological recovery with likely permanent cognitive and motor deficits. His code status was subsequently changed to do not resuscitate (DNR) with no further escalation of care. The patient continued to deteriorate throughout the night, became asystolic, and expired.

## Discussion

The most commonly used classification system for an acute aortic dissection is the Stanford classification system. A Stanford type A dissection involves any part of the ascending aorta. A Stanford type B dissection does not involve the ascending aorta, rather only the descending thoracic aorta distal to the left subclavian artery. It is important to note that a Stanford type A dissection may involve the descending aorta as well, but ascending aortic involvement is the sole determinant of the type of dissection [2].

High-risk conditions associated with an aortic dissection include, but not limited to:

- Systemic hypertension (most significant predisposing factor)
- Collagen-vascular disorders (Ehlers-Danlos syndrome, Marfan’s syndrome, annuloaortic ectasia)
- Pre-existing aortic aneurysm
- Bicuspid aortic valve
- Coronary artery bypass grafting, cardiac catheterization
- Turner’s syndrome
- Aortic coarctation
- Inflammation secondary to vascular diseases (rheumatoid arthritis, Takayasu arteritis, giant cell arteritis, and syphilitic aortitis)
- Chest trauma secondary to an acute deceleration

The International Registry of Acute Aortic Dissection (IRAD) review, as originally published in the Journal of the American Medical Association, involved 12 international referral centers and 464 enrolled patients with an aortic dissection and became the largest registry in the world on aortic dissection [3]. The classic description of pain has often been described as an abrupt onset of a ripping or tearing sensation; however, this is only present about 50% of the time. The most common description is rather a knife/sharp stabbing pain at about 68% of the time [1, 3]. According to the study, chest pain was most commonly found in patients with a Stanford type A dissection (83% vs 71%), while abdominal pain and back pain were most commonly found in patients with a Stanford type B dissection (43% vs 22%) [1, 3].

The vitals and clinical manifestations can be vast and depend on the extent of the dissection, location, and structures involved. Hypertension on arrival to the ED is seen in about 70% of Stanford type B dissections, while only present in 35% of Stanford type A dissection [1, 3]. Those patients that present with hypotension have disruption/prolapse of the aortic valve, resulting in severe aortic regurgitation (diastolic murmur associated with severe chest pain, hypotension. and a wide pulse-pressure), and/or extension into the pericardial space (cardiac tamponade) [1, 3].

A pulse deficit is often mentioned in the literature (a variation > 20 mmHg when comparing both arms); however, in patients with a Stanford type A dissection, a pulse deficit only occurs in 30% of patients, and 21% in those with a Stanford type B dissection [1, 4]. Overall, those patients that did present with a pulse deficit had concomitant neurologic deficits, had hypotension, and were in a coma [1, 5].

Other clinical manifestations include, but not limited to:

- Focal neurologic deficits (secondary to propagation to involve the carotid arteries and will mimic a cerebrovascular accident)
- Hoarseness (result of the left recurrent laryngeal nerve becoming compressed)
- Paraplegia (secondary to ischemia to the spinal cord)
- Horner’s syndrome (result of compression of the superior cervical sympathetic ganglion)
- Coronary ischemia on electrocardiography (ECG) (1 in 100 patients and associated most often with an inferior wall distribution)
- Multi-system organ failure (secondary to involvement of multiple abdominal aortic branches) [6].

The diagnosis is suspected clinically based on the presence of high-risk clinical features and is confirmed on CT angiography demonstrating a dissection flap [1]. In the IRAD review, 63% of patients with a Stanford type A dissection had mediastinal widening on plain chest radiography while 56% of patients with a Stanford type B dissection had mediastinal widening [3]. A widened mediastinum has been defined as > 8 cm at the aortic knob on supine radiography, > 6 cm on upright radiography, or a mediastinum/chest width ratio > 0.25 [7, 8].

Utilization of a d-dimer is often mentioned in the literature in relation to an aortic dissection. A d-dimer reflects an activation of the coagulation cascade by tissue factor that becomes exposed within the aortic media when an intimal tear occurs [1]. A systematic review involving seven studies utilizing a d-dimer (< 500 ng/mL) to screen patients for a dissection resulted in a sensitivity of 97%, specificity of 56%, and a negative predictive value of 96% [1]. While a widely used cutoff has been a d-dimer < 500 ng/mL for excluding dissection, one large multicenter study incorporating 1850 patients with an aortic dissection found that 8% of them had a negative d-dimer [1]. It is for this reason that the guidelines, as published in the Annals of Emergency Medicine, do not recommend the use of d-dimer in the evaluation of an aortic dissection [9].

A Stanford type A dissection is considered a surgical emergency, while a Stanford type B dissection in stable patients can be treated medically. Without treatment, half of all patients with a Stanford type A dissection will die within 48 h, and even with rapid surgical treatment, the mortality approaches 25% [2]. Medical management consists of reversing anticoagulation, providing analgesia, maintaining a systolic blood pressure of < 110 mmHg and a heart rate < 60 beats/min (decreases extension of the dissection flap) [4, 5]. Common medications utilized for this include beta-blockers and vasodilators. The current recommendation is to control the heart rate before the blood pressure. Beta-blockers are excellent first-line options since they decrease aortic wall tension and heart rate. Examples include esmolol (cardio-selective beta-1 blocker) and labetalol (non-selective beta-blocker and alpha-1 blocker). In patients that have a contraindication to beta-blockers (i.e., severe aortic regurgitation), diltiazem is a first-line option. If beta-blockers are ineffective or an additional medication is needed, vasodilators such as nicardipine or nitroprusside are recommended. As a caution, reflex tachycardia must be suppressed and shear forces from an increased heart rate must be avoided. It is for this reason that vasodilators are not recommended unless a beta-blocker is initiated first [10, 11].

## Conclusion

Our case presentation provides physicians a brief up to date literature review on aortic dissection. This case became an unexpected presentation as the patient’s dissection rapidly worsened from his initial onset of isolated chest pain to his shortness of breath, stroke-like symptoms, and worsening lower extremity weakness throughout his ED stay. Despite early recognition and treatment, our patient only survived to day 5.

## Data Availability

Data sharing is not applicable to this article as no datasets were generated or analyzed during the current study.

## Abbreviations

CCRT: Continuous renal replacement therapy
CT: Computed tomography
DNR: Do not resuscitate
ECG: Electrocardiography
ED: Emergency department
EMS: Emergency medical services
IRAD: International Registry of Acute Aortic Dissection

## References

[1] Black J, Manning W. Clinical features and diagnosis of acute aortic dissection. Uptodate. 2018; https://www.uptodate.com/contents/clinical-features-and-diagnosis-of-acute-aortic-dissection.

[2] Borloz, M. “Thoracic Aortic Dissection.” 2016. Society of Academic Emergency Medicine. https://saem.org/cdem/education/online-education/m4-curriculum/group-m4-cardiovascular/thoracic-aortic-dissection.

[3] Hagan, P, Nienaber, C, Isselbacher, E, Bruckman, D, Karavite, D, Russman, P, et al. “The International Registry of Acute Aortic Dissection (IRAD): new insights into an old disease.” https://www.ncbi.nlm.nih.gov/pubmed/10685714. 10.1001/jama.283.7.897 10685714

[4] Diercks D (2015). Clinical policy: critical issues in the evaluation and management of adult patients with suspected acute nontraumatic thoracic aortic dissection. Ann Emerg Med.

[5] Imamura H, Sekiguchi Y, Iwashita T (2011). Painless acute aortic dissection. Diagnostic, prognostic and clinical implications. Circ J.

[6] Helman, A. “How to diagnose aortic dissection without breaking the bank.” 2017. ACEP Now. https://www.acepnow.com/article/diagnose-aortic-dissection-without-breaking-bank/

[7] Meyer C, Engelbrecht A. Traumatic aortic dissection presenting with respiratory arrest. 2015;5(1):e5–7 https://www.sciencedirect.com/science/article/pii/S2211419X14001359?via%3Dihub.

[8] Woodring John H., Dillon Marcus L. (1984). Radiographic Manifestations of Mediastinal Hemorrhage from Blunt Chest Trauma. The Annals of Thoracic Surgery.

[9] Diercks D, Promes S, Schuur J (2015). Clinical policy: critical issues in the evaluation and management of adult patients with suspected acute non-traumatic thoracic dissection. Ann Emerg Med.

[10] Kodama K, Nishigami K, Sakamoto T, Sawamura T, Hirayama T, Musmi H (2008). Tight heart rate control reduces secondary adverse events in patients with type B acute aortic dissection. Circ J.

[11] Hebballi R, Swanevelder J (2009). Diagnosis and management of aortic dissection. Contin Educ Anaesth Crit Care Pain.

